# Impact of residual thymic symptoms in quality of life in bipolar patients in euthymia

**DOI:** 10.1192/j.eurpsy.2021.514

**Published:** 2021-08-13

**Authors:** S. Elleuch, R. Sellami, R. Ouali, I. Feki, J. Masmoudi

**Affiliations:** 1 Psychiatrie “a” Department, Hedi Chaker Hospital University -Sfax - Tunisia, sfax, Tunisia; 2 Psychiatry “a” Department, Hedi Chaker Hospital University -Sfax - Tunisia, sfax, Tunisia

**Keywords:** euthymia, bipolar disorder, FAST scale, thymic residual symptoms

## Abstract

**Introduction:**

Several studies have shown that residual mood symptoms affect the psychosocial functioning of patients with bipolar disorder (BD) in euthymia.

**Objectives:**

To evaluate specific areas of functioning in this population and to explore the relationship with residual mood symptoms.

**Methods:**

This is a descriptive and analytical cross-sectional study including patients with BD (DSM V) in euthymia followed on ambulatory basis to the Mood Disorders Unit of the Psychiatry A Department at Hedi Chaker Hospital in Sfax between January and April 2019. Patients were considered euthymic if they scored below 7 on the Young Mania Assessment Scale (YMRS) and under 8 on the Hamilton Depression scale (HDRS-17). Residual manic and depressive mood symptoms were assessed using YMRS and HDRS-17. The Short Function Evaluation Test (FAST) was used to evaluate the overall and specific functionning domains.The alteration of the domain-specific functioning is defined by the following thresholds: autonomy>1, professional functioning>1, cognitive functioning>2, financial problems>1, interpersonal relations>3 and leisure time>3.

**Results:**

We recruited 62 patients with a mean age of 45.65 years (SD = 13.3) and a sex ratio 1.13.The medians of YMRS and HDRS scores were respectively 2[0-5] and 2[0-7]. Global functionning impairment was observed in 85.5% of patients. Marked impairment of professional and cognitive functioning was observed in 98.4% and 77.4%, respectively. Alteration of the relational sphere was significantly more frequent in patients with residual depressive symptoms (p=0.009); impairment of autonomy was significantly more frequent in subjects with manic residual symptoms (p=0.005).

**Conclusions:**

Residual symptoms should be considered as specific targets of treatment to improve functioning.

